# Editorial: The relevance of molecular mechanisms in HIV-1 latency and reactivation from latency

**DOI:** 10.3389/fcimb.2023.1190867

**Published:** 2023-04-03

**Authors:** Alexander O. Pasternak, Olivier Rohr, Carine Van Lint, Anna Kula-Pacurar

**Affiliations:** ^1^ Laboratory of Experimental Virology, Department of Medical Microbiology, Amsterdam University Medical Center, University of Amsterdam, Amsterdam, Netherlands; ^2^ Laboratoire de Dynamique des Interactions Hôte-Pathogènes EA7292, Université de Strasbourg, Schiltigheim, France; ^3^ Institut Universitaire de Technologie Louis Pasteur, Université de Strasbourg, Schiltigheim, France; ^4^ Service of Molecular Virology, Department of Molecular Biology, Université Libre de Bruxelles, Gosselies, Belgium; ^5^ Virogenetics Laboratory of Virology, Malopolska Centre of Biotechnology, Jagiellonian University, Krakow, Poland

**Keywords:** HIV-1, HIV-1 latency, HIV-1 reservoir, HIV-1 latency reversal, HIV-1 persistence

Despite the remarkable efficacy of antiretroviral therapy (ART) that dramatically reduced morbidity and mortality among people with HIV-1 (PWH), the infection is uncurable due to the persistence of the viral reservoir in a small population of latently infected cells. The latent reservoir consists of stably integrated, replication-competent intact proviruses that are repressed by a plethora of interconnected cellular and viral silencing mechanisms operating at epigenetic, transcriptional, and post-transcriptional levels (Dutilleul et al., 2020; Verdikt et al., 2022). Moreover, the integration site of the provirus, its genomic location and spatial positioning in the nucleus, as well as the transcriptional program of the host cell, influence establishment and maintenance of HIV-1 latency (Lichterfeld et al., 2022). The “shock-and-kill” strategy that uses latency-reversing agents (LRAs) to reactivate the virus for the subsequent elimination of the reservoir by immune clearance or viral cytopathic effects is one of the most explored HIV-1 curative strategies (Kula-Pacurar et al., 2021; Rodari et al., 2021). However, so far, the “shock-and-kill” clinical trials have been rather disappointing with no significant impact on the reservoir size. Heterogeneity of the latent reservoirs and the complexity of epigenetic, transcriptional, and post-transcriptional molecular mechanisms regulating HIV-1 latency likely contribute to the limited success of this strategy (Ait-Ammar et al.). Therefore, a better understanding of molecular mechanisms underlying HIV-1 latency and reactivation that are driven by virus-host interactions is pivotal in designing more potent approaches to reach a cure.

In this Research Topic, we welcomed reviews and original research articles that explored molecular mechanisms contributing to HIV-1 latency and reactivation, with a special emphasis on the virus-host interplay.

Among HIV-1 cure therapeutic targets, the Akt pathway has been explored and reviewed by Pasquereau and Herbein. The protein kinase B or Akt has been reported to regulate cell survival upon viral infection, but also HIV-1 entry and HIV-1 gene transcription. The upstream regulator of Akt, Phosphoinositide 3-kinase (PI3k), transduces extracellular signals to stimulate Akt and regulate its downstream targets such as mTOR. The authors review how the activation of the PI3K/Akt pathway favours HIV-1 entry as well as the establishment and the persistence of the latently infected reservoirs. They report that Akt regulates HIV-1 gene expression transcriptionally, post-transcriptionally, and epigenetically. The PI3K/Akt pathway differentially modulates pro-inflammatory responses and immune cell activation in T cells, NK cells and macrophages. The authors suggest anti-PI3K/Akt drugs and therapeutic strategies to limit the viral spread, modulate immune stimulations and limit latently infected reservoirs. Finally, a new “Block and clear” strategy is proposed to “counterAKT HIV” and cure the infected individuals.

Randolph et al. provide an excellent up-to-date overview of advances towards understanding the role of Krüppel-associated box (KRAB)-associated protein 1 (KAP1: also named TRIM28 or TIF1β), which has been extensively studied in the past three decades in the context of transcriptional regulation of endogenous retroviruses, DNA and RNA viruses, including HIV-1 and diverse cellular programs such as immune responses or development. Importantly, Randolph et al. outline in detail the controversial pleiotropic roles of this factor in HIV-1 gene expression and latency, as it can function either as activator or repressor of the viral transcription. Authors further propose a paradigm in which KAP1 regulates a “switch” from repression to activation of transcription. Understanding of the molecular details determining the KAP1-mediated switch will open potential therapeutic opportunities to target HIV-1 infection or latency. These future findings can be crucial in designing new ways to reactivate or repress silent virus.

HIV-1 reservoirs are believed to persist primarily by infected cell longevity and clonal proliferation (Chomont et al., 2009; Pasternak and Berkhout, 2023). Previous studies reported a significant enrichment of proviral integrations in genes that are involved in cellular proliferation and survival in cells from ART-treated PWH (Maldarelli et al., 2014; Wagner et al., 2014). In theory, such integrations can lead to clonal expansion of the infected cell, which, in turn, would favour viral persistence. One mechanism by which a provirus may modulate host gene expression is aberrant splicing that produces virus-host chimeric mRNAs. Such chimeras have the potential to alter cellular gene expression and could even lead to proliferation of the host cell (Cesana et al., 2012; Moiani et al., 2012). Lee et al. report detection of such chimeric virus-host mRNAs that were generated by aberrant splicing. Using target enrichment coupled to the Illumina Mi-Seq and PacBio RS II platforms, they show that 3’ LTR activation is frequent in latently infected cells from both the CCL19-induced primary cell model of HIV-1 latency as well as *ex vivo* samples from ART-treated PWH. In both systems, they detected several chimeric species that were generated *via* activation of a cryptic splice donor site in the 5’ LTR of HIV-1. In addition, their results argue that production of HIV-1 proteins is not favoured from chimeric transcripts and thus these transcripts are unlikely to perturb HIV-1 latency.

While viral latency is generally defined as a reversible state of non-productive infection, which does not necessarily imply lack of viral gene expression, the latent HIV-1 reservoir has been traditionally viewed as integrated proviruses that are transcriptionally silent but can be reactivated to transcribe viral RNA and produce infectious virus. Accordingly, most of current LRAs function by stimulating HIV-1 transcription. However, HIV-1 latency can be regulated not only at the transcriptional but also at multiple post-transcriptional levels (Yukl et al., 2018). More than a decade ago, the “continuum of latency” was proposed, with barriers to productive infection at different stages of the viral replication cycle in different populations of latently infected cells, and the concept of the active HIV-1 reservoir was introduced to describe latently infected cells that are actively transcribing HIV-1 RNA but do not produce infectious virus particles (Pace et al., 2011; Pasternak et al., 2013). Different RNA processing pathways likely play an important role in HIV-1 persistence (Pasternak and Berkhout, 2021) and therefore might present an opportunity for therapeutic targeting as part of HIV-1 cure efforts. In their elegantly titled review “HibeRNAtion: HIV-1 RNA Metabolism and Viral Latency”, Crespo et al. discuss the various steps of HIV-1 RNA processing, such as splicing, nucleocytoplasmic transport, RNA surveillance and degradation, epitranscriptomic RNA modifications, or translation, and describe how post-transcriptional control of HIV-1 and its dysregulation can contribute to latency. They also speculate about possible therapeutic approaches in targeting these post-transcriptional steps of HIV-1 RNA processing and their potential implications in HIV-1 cure research.

In summary, the articles presented in this Research Topic provide a useful overview of molecular mechanisms of HIV-1 latency and propose novel approaches to target them.

## Author contributions

All authors listed have made a substantial, direct, and intellectual contribution to the work and approved it for publication.
