# Corrigendum: Editorial: The relevance of molecular mechanisms in HIV-1 latency and reactivation from latency

**DOI:** 10.3389/fcimb.2023.1213356

**Published:** 2023-05-11

**Authors:** Alexander O. Pasternak, Olivier Rohr, Carine Van Lint, Anna Kula-Pacurar

**Affiliations:** ^1^ Laboratory of Experimental Virology, Department of Medical Microbiology, Amsterdam University Medical Center, University of Amsterdam, Amsterdam, Netherlands; ^2^ Laboratoire de Dynamique des Interactions Hôte-Pathogènes EA7292, Université de Strasbourg, Schiltigheim, France; ^3^ Institut Universitaire de Technologie Louis Pasteur, Université de Strasbourg, Schiltigheim, France; ^4^ Service of Molecular Virology, Department of Molecular Biology, Université Libre de Bruxelles, Gosselies, Belgium; ^5^ Virogenetics Laboratory of Virology, Malopolska Centre of Biotechnology, Jagiellonian University, Krakow, Poland

**Keywords:** HIV_-1_, HIV_-1_ latency, HIV_-1_ reservoir, HIV_-1_ latency reversal, HIV_-1_ persistence

**Incorrect Funding**

In the published article, there was an error in the Funding statement. The Funding section misses the funding agency for AKP. The correct Funding statement appears below.

FUNDING

AOP acknowledges funding from the Dutch Medical Research Council (ZonMw), grant no. 09120011910035. CVL and OR acknowledge funding from the Belgian National Fund for Scientific Research (F.R.S-FNRS, Belgium); the French INSERM agency “ANRS/Maladies infectieuses émergentes”; the “Télévie” program of the F.R.S.-FNRS; ViiV Healthcare; the “Fondation Roi Baudouin”; the Internationale Brachet Stiftung (IBS); the Walloon Region (“Fonds de Maturation”); The “Amis des Instituts Pasteur à Bruxelles”, asbl.; the University of Brussels (ULB - Action de Recherche Concertée (ARC) grant); the NEAT (European AIDS Treatment Network) program; the University of Brussels (Action de Recherche Concertée ULB grant); the Marie Skłodowska-Curie COFUND action; and the European Union’s Horizon 2020 research and innovation program under grant agreement No 691119-EU4HIVCURE-H2020-MSCA-RISE-2015. CVL is “Directrice de Recherches” of the F.R.S-FNRS. The laboratory of CVL is part of the ULB-Cancer Research Centre (U-CRC) (Faculty of Medicine, ULB). AKP acknowledges funding from the National Science Centre, Poland (Sonata Bis Grant UMO2018/30/E/NZ1/00874).

The authors apologize for this error and state that this does not change the scientific conclusions of the article in any way. The original article has been updated.

